# Hysteresis data of planar perovskite solar cells fabricated with different solvents

**DOI:** 10.1016/j.dib.2017.11.064

**Published:** 2017-11-22

**Authors:** You-Hyun Seo, Eun-Chong Kim, Se-Phin Cho, Seok-Soon Kim, Seok-In Na

**Affiliations:** a Professional Graduate School of Flexible and Printable Electronics and Polymer Materials Fusion Research Center, Chonbuk National University, Jeonju-si, Jeollabuk-do 561-756, Republic of Korea; b Department of Nano & Chemical Engineering, Kunsan National University, 290-2, Miryong-dong, Gunsan-si, Jeollabuk-do 573-701, Republic of Korea

## Abstract

In this data article, we introduced the hysteresis of planar perovskite solar cells (PSCs) fabricated using dimethylformamide (DMF), gamma-butyrolactone (GBL), methyl-2-pyrrolidinone (NMP), dimethylsulfoxide (DMSO), DMF-DMSO, GBL-DMSO and NMP-DMSO as perovskite precursor solutions according to different scan directions, sweep times, and current stability. The hysteresis analyses of the planar PSCs prepared with a glass-ITO /NiO_X_/perovskite /PC_61_BM/BCP/Ag configuration were measured with Keithley 2400 source meter unit under 100 mW/cm^2^ (AM 1.5 G). The data collected in this article compares the hysteresis of PSCs with different solvents and is directly related to our research article “High-Performance Planar Perovskite Solar Cells: Influence of Solvent upon Performance” (You-Hyun Seo et al., 2017 [1]).

**Specifications Table**

**Table:** 

Subject area	*Electrical Engineering*
More specific subject area	*Perovskite Solar Cells*
Type of data	*Figure*
How data was acquired	*Keithley 2400 source meter unit under 100 mW/cm*^*2*^*(AM 1.5 G)*
Data format	*Analyzed*
Experimental factors	*Current density-voltage (J-V) scans: Different sweep directions, different scan time, current stability*
Experimental features	*Forward and reverse bias range: 1.5 V to -0.2 V and -0.2 V to 1.5 V*
	*Dwell time range: 0*–*500* *ms*
	*Current stability: The 301 scan points were recorded during ~ 80 s scan times at each maximum-voltage.*
Data source location	*Chonbuk National University, Jeonju-si, Jeollabuk-do, 561–756, Republic of Korea*
Data accessibility	*Data is with this article.*

**Value of the data**

• The data article presents the variations of hysteresis curves in PSCs with DMF, GBL, NMP, DMSO, DMF-DMSO, GBL-DMSO, and NMP-DMSO.
• Different sweep directions, different scan times, and current stability characteristics of PSCs with different solvents would be useful for insight of hysteresis behavior.
• These data can provide better understanding for research into the influence of solvent in planar PSCs.

## 1 Data

**Fig. 1:**
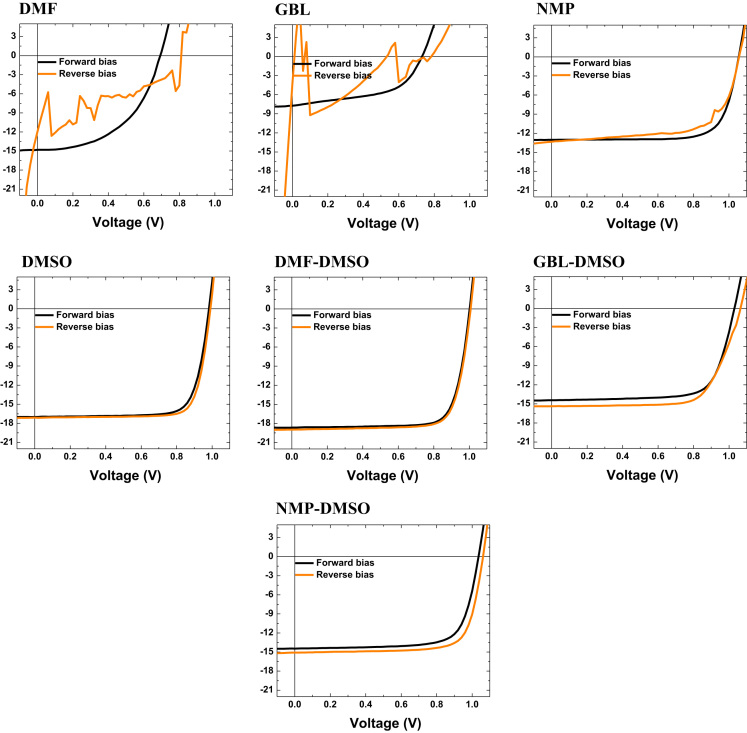
J-V curves of PSCs with different solvents in different sweep directions.

**Fig. 2:**
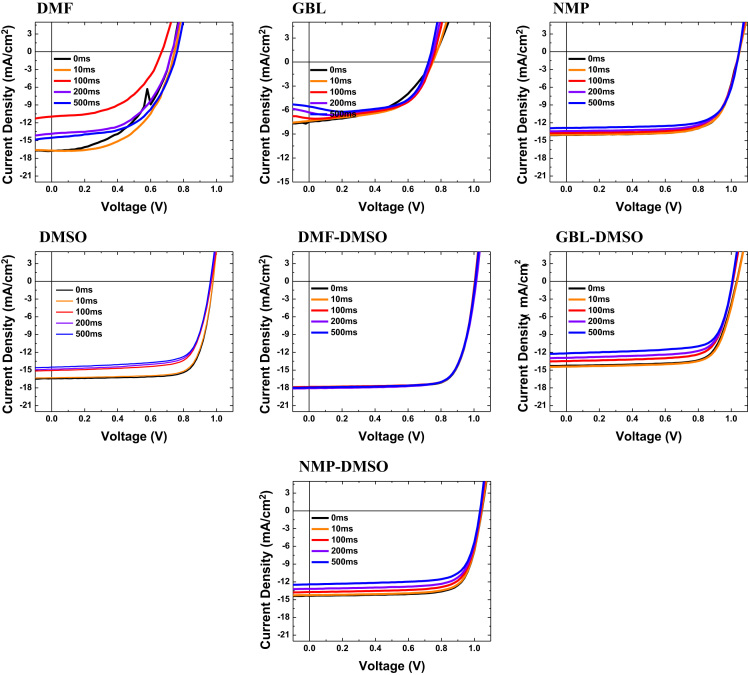
J-V curves of PSCs with different solvents in different scan times.

**Fig. 3:**
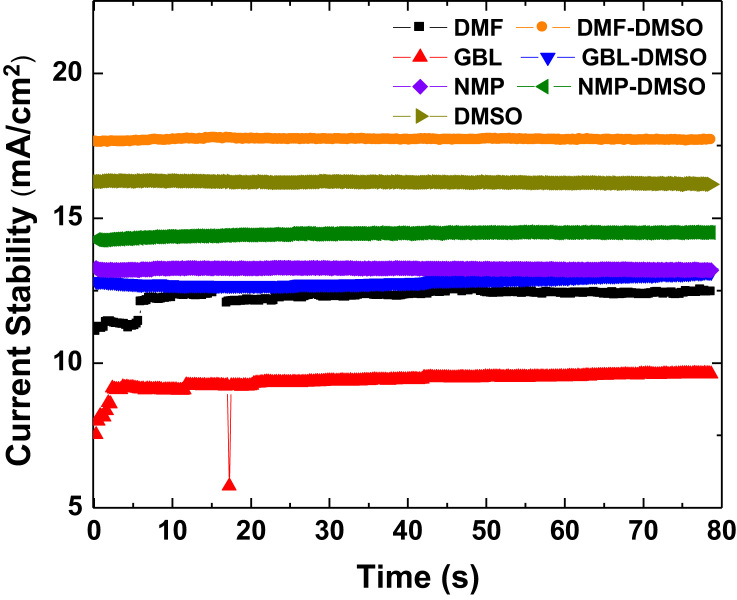
Current stability data of perovskite devices with different solvents measured with different times under the maximum voltage point in the illumination.

We investigated the hysteresis of PSCs fabricated using different solvents according to different scan directions, sweep times, and current stability [2], [3], as shown in Fig. 1, Fig. 2, Fig. 3. Previous reports suggested that such hysteresis could be induced by the ion migration, ferroelectricity, charge trapping or detraining, and so on [2], [4]. From the hysteresis plot, it can be confirmed that most of perovskite devices showed the hysteresis, while the PSCs with DMF-DMSO did not provide any distinct hysteresis curves and showed highest current flows, thus suggesting that the DMF-DMSO can be a better choice for preparing better-performance planar-based perovskite solar cells.

## 2 Experimental design, materials and methods

Seven different perovskite films were prepared for planar PSCs [1]. For the hysteresis analyses, each J-V curve was recorded under 100 mW/cm^2^ illumination at AM (air mass) 1.5 G condition with a Keithley 2400 instrument calibrated with a Si solar cell (SRC 1000 TC KG5 N, VLSI Standards, Inc). For accurate comparisons, the collected J-V curve was chosen as close to statistical analysis of each PSC [1]. These hysteresis data may provide useful information for the influence of solvent in planar PSCs.
